# Intrusive Memories of Distressing Information: An fMRI Study

**DOI:** 10.1371/journal.pone.0140871

**Published:** 2016-09-29

**Authors:** Eva Battaglini, Belinda Liddell, Pritha Das, Gin Malhi, Kim Felmingham, Richard A. Bryant

**Affiliations:** 1 University of New South Wales, Sydney, Australia; 2 University of Sydney, Sydney, Australia; 3 University of Melbourne, Melbourne, Australia; University of Cambridge, UNITED KINGDOM

## Abstract

Although intrusive memories are characteristic of many psychological disorders, the neurobiological underpinning of these involuntary recollections are largely unknown. In this study we used functional magentic resonance imaging (fMRI) to identify the neural networks associated with encoding of negative stimuli that are subsequently experienced as intrusive memories. Healthy partipants (N = 42) viewed negative and neutral images during a visual/verbal processing task in an fMRI context. Two days later they were assessed on the Impact of Event Scale for occurrence of intrusive memories of the encoded images. A sub-group of participants who reported significant intrusions (n = 13) demonstrated stronger activation in the amygdala, bilateral ACC and parahippocampal gyrus during verbal encoding relative to a group who reported no intrusions (n = 13). Within-group analyses also revealed that the high intrusion group showed greater activity in the dorsomedial (dmPFC) and dorsolateral prefrontal cortex (dlPFC), inferior frontal gyrus and occipital regions during negative verbal processing compared to neutral verbal processing. These results do not accord with models of intrusions that emphasise visual processing of information at encoding but are consistent with models that highlight the role of inhibitory and suppression processes in the formation of subsequent intrusive memories.

## Introduction

Involuntary intrusive memories, thoughts or images are often distressing symptoms that occur in a wide variety of clinical disorders, including posttraumatic stress disorder (PTSD) [1], depression [2], health anxiety [3], agoraphobia [4], and social anxiety [5]. Despite their prevalence and clinical impact, the neural mechanisms underlying intrusions are not well understood.

Several psychological models have been developed to account for the formation and experience of intrusive memories. These attribute intrusions to factors such as: disruption of consolidation processes that facilitate the incorporation of experiences into autobiographical memory [6, 7], over-suppression and monitoring of difficult memories or emotionally-salient events [8, 9], association of intrusive memory with internal or external triggers that were conditioned with the intrusive memory [10], or that involuntary memories share the same underlying episodic memory system as voluntary memories, but are recalled associatively, initiated by situational cues [11].

A prevailing model of PTSD suggests that data-driven or perceptually based encoding leads to a greater likelihood of memories later becoming intrusive, while conceptual or verbally-based encoding is thought to be protective against later development of intrusions [12]. Intrusions may occur when a perceptually based memory is not properly linked with its corresponding verbally-based memory. This can lead to the perceptual memory being retrieved out of its proper autobiographical and semantic context, appearing in a vivid and distressing form [7]. Prospective studies have found that self-reported visual or data-driven processing at the time of encoding memories of a traumatic event predicts subsequent PTSD symptoms [13, 14]. Similar results have been reported following stressful life events [15]. Relatedly, there is evidence in healthy participants that data-driven processing leads to more intrusions than conceptual processing [16, 17]. It has also been found that completing a competing verbal task while viewing a trauma film leads to increased intrusions [18]. These findings suggest that greater availability of visual memory resources to process and consolidate trauma memories can lead to the formation of intrusive memories.

There has been little neuroimaging research to date that investigates the neural bases of intrusions, or the role of visual and verbal processing in the encoding of intrusive memories. However, predictions regarding the brain regions involved in the encoding of intrusions may be drawn from the autobiographical and emotional memory imaging literature. This field is relevant since intrusions often take the form of repeated, unwanted automatic retrievals of significant and emotional autobiographical memories. Autobiographical and emotional memory both involve multiple and complex neural systems, with activity in varying brain regions being seen across studies [19]. In previous neuroimaging studies of autobiographical memory, different brain regions have been associated with specific components of the autobiographical memory retrieval process. The lateral prefrontal cortex has been associated with search processes, the ventromedial prefrontal cortex with monitoring processes, and the medial prefrontal cortex (mPFC) with self-referential processes, while the amygdala and visual cortex have been implicated in emotion and vividness of the memory respectively [20]. When specifically considering the experience of unpleasant autobiographical memories, a network involving the amygdala, anterior cingulate cortex (ACC) and medial prefrontal cortex has been implicated [21], and amygdala activity at encoding has been found to be positively correlated with the ability to retrieve emotional memories [22]. Other medial temporal lobe (MTL) structures, including the hippocampus and parahippocampal gyrus, also appear to be involved. The hippocampus functions to enable contextual fear learning [23], while the posterior parahippocampal gyrus has been implicated in the perceptual processing, encoding and retrieval of memories for scenes and places [24], as well as emotional memory encoding [25]. Furthermore, systems responsible for response inhibition and cognitive control may be recruited if individuals attempt to inhibit their automatic reactions to an intrusive memory, especially if the content is negative. Cognitive control of emotion and attentional responses appears to rely on a network of cortical regions, including ACC, mPFC, orbitofrontal cortex (OFC), ventrolateral prefrontal cortex (vlPFC) and dorsolateral prefrontal cortex (dlPFC) [26].

Research to date into the neural correlates of autobiographical and emotional memory has largely neglected to distinguish between memories that are experienced intentionally vs involuntarily (intrusions), with only 3 studies to date. In a PET study, healthy participants were initially presented with images, followed by a second presentation of the same images where a cue word was aurally presented along with each image, and participants generated a sentence which included the word and described the content of the image [27]. This task was designed to ensure explicit memory for initial the image-word cue association. During PET scanning, participants were instructed to recall the picture associated with the original cue word (voluntary condition), or used a button press to make a semantic categorization of the cue word (involuntary condition–intended to model intrusions of the encoded images). Participants were not informed that cue words during the involuntary condition were intended to provoke involuntary memories of the associated images, but indicated after scanning whether or not they had recalled the images associated with these cues. This study found that, compared to a control condition, both voluntary and involuntary recall were associated with regional cerebral blood flow (rCBF) increases in the posterior cingulate gyrus, left precuneus, and right parahippocampal gyrus. Additionally, voluntary recall was specifically associated with increased rCBF in the right dlPFC and left precuneus compared to involuntary recall, and involuntary recall was associated with increased rCBF in the left dlPFC. However, this study did not distinguish between involuntary memories of emotional and neutral stimuli. Since intrusions generally take the form of thoughts or memories with strong emotional content, this study could be seen as lacking an important aspect of intrusions as they occur in a clinical context.

The second study investigated the neural bases of intrusions utilized during a trauma film paradigm [28]. Healthy participants viewed a film that included both negative and neutral scenes while undergoing a functional Magnetic Resonance Imaging (fMRI) scan, and completed an intrusion diary for seven days post scan. The encoding of negative scenes that were associated with subsequent intrusions was compared to the negative scenes that did not become intrusive, as well as to neutral scenes. The encoding of subsequent intrusive negative content was associated with increased activation in the amygdala, ventral occipital cortex, rostral ACC, inferior frontal gyrus and medial temporal gyrus. These regions have been broadly associated with emotional processing, mental imagery, threat processing, and flagging of salient events to be remembered. However, this study did not investigate the effects of different modes of processing at encoding upon the later development of intrusions, something which the current study seeks to examine.

A third study investigated the neural bases of flashbacks experienced by participants with PTSD [29]. Flashbacks were triggered during an fMRI scan using personalized trauma-relevant word cues. The experience of flashbacks, compared to ordinary episodic trauma memories, was associated with increased activity in the insula, motor (precentral gyrus, supplementary motor area) and sensory areas (occipital cortex), as well as decreased activation in the midbrain, parahippocampal gyrus, precuneus and posterior cingulate cortex. These findings suggest that the neurocircuitry underpinning flashbacks in PTSD is distinct from autobiographical memory systems, involves increases in dorsal visual processing and results in decreased activity in regions associated with contextualizing memories.

To investigate the neural networks involved in encoding intrusive memories under visual and verbal processing conditions, we used a modified version of a paradigm previously employed in fMRI studies [30]. The original study aimed to identify neural response patterns during visual and verbal processing of emotional stimuli. Results showed that visual (or perceptual) processing involved amygdala activation, while verbal (or cognitive) evaluation showed an attenuation of amygdala activation, with a concomitant increase in prefrontal cortex (PFC) and ACC activation. Given the hypothesized importance of the mode of encoding in the formation of intrusive memories, activation within these distinct pathways for visual vs. verbal encoding may be critical to the formation of intrusive memories. Thus, the use of this paradigm (including highly negative images which may be likely to be recalled intrusively) may help to elucidate the role of mode of processing in the formation of intrusions.

The current study used a similar experimental paradigm to investigate the effect of visual and verbal processing on the encoding of intrusive memories. We hypothesized that intrusions following the viewing of negative images will be associated with increased activation in the amygdala and MTL structures, which are associated with encoding of emotional memories. Areas such as the ACC and mPFC could also be involved, if the negative images are unpleasant enough to provoke inhibition of emotional responses. It was expected, in line with previous research, that the subsequent experience of intrusions would be associated with enhanced activity in these regions, as well as the inferior frontal gyrus, insula, motor and sensory regions. It was further hypothesized that intrusion-related activation in the above regions would be associated more with the visual processing condition than the verbal processing condition, and that greater activation in the areas mentioned above will be seen during visual processing than verbal processing conditions. In line with Hariri et al.’s visual/verbal processing study, the visual processing conditions may be particularly associated with amygdala activity, while verbal processing conditions are expected to involve PFC and ACC activation, while showing reductions in amygdala activation.

## Method

### Participants

Forty-two participants took part in the study (27 female, 15 male; mean age 20.1 years, SD = 3.0). Participants were healthy undergraduate psychology students from the University of New South Wales, who participated in return for partial course credit.

At recruitment, participants were excluded if they had: a current diagnosis or history of psychological disorder; history of serious brain injury or loss of consciousness for more than ten minutes; history of stroke or neurological disorder; severe non-correctable impairment of vision; impairment of hearing or hand movement; and current or previous heavy consumption of alcohol and other drugs (e.g. marijuana, heroin, cocaine, amphetamines). This study was approved by the Northern Sydney Local Health District and University of New South Wales human research ethics committees, and all participants gave written informed consent prior to participating.

### Measures

#### Pre-scanning questionnaires

Before undergoing the scan, participants completed the following self-report questionnaires: Beck Depression Inventory- Second Edition (BDI-II [31]) to assess depression symptoms over the previous fortnight; State-Trait Anxiety Inventory (STAI [32]) trait anxiety subscale; Impact of Event Scale (IES [33]) to assess the extent to which participants experienced intrusions relating to any previously experienced negative event over the week prior to scanning; and the Vividness of Visual Imagery Questionnaire (VVIQ [34]) to assess individual differences in vividness of mental imagery.

#### Post-scanning questionnaire

This four item questionnaire was used to investigate participants’ general perceptions of the stimuli from a third person viewpoint. The instructions read as follows: ‘Thanks for looking at all these images. Before we finish, I would like to get a sense of how you think people would generally rate these sorts of images. In the final section of the experiment, you were asked to look at some neutral objects and some disfigured bodies. Can you rate below how you think *most* people would rate these images on some dimensions:’ Following these instructions, questions included: ‘How negative would most people find these?’ and ‘How positive would most people find these?’, with responses provided on a 10-point Likert-type scale (1 = *Not at all*, 10 = *Extremely*). Negative and neutral images were rated separately.

#### Follow-up questionnaire

To measure the subsequent experience of intrusions, a reduced version of the Impact of Event Scale (IES) was administered two days after scanning. The measure consisted of 4 items from the IES to index intrusive recollections of the images presented during the scan, specifically: ‘Any reminder brought back feelings of it’, ‘Other things kept making me think about it’, ‘I thought about it when I didn’t mean to’, and ‘Pictures about it popped into my mind’), with responses provided on a five-point Likert-type scale (0 = *Not at all*, 4 = *Extremely*). This subset of IES items was selected on the basis that they were the most directly relevant to the experience of intrusions related to the encoded images. Instructions for the questionnaire were as follows: ‘Think about the pictures you were shown when you came to the experiment a few days ago. In the last section of the experiment, you were shown some pictures of damaged bodies or neutral objects. Can you briefly describe some examples of these pictures?’ Space was provided for participants to list some examples of each type stimulus content under the headings ‘Damaged bodies’ and ‘Neutral objects’, allowing for a manipulation check, ensuring that participants were able to distinguish between the neutral and negative stimuli. Instructions then continued: ‘We are interested in whether any of those pictures have popped into your mind at any time after the experiment. We want you to answer some questions about the 2 groups of images. First of all, think about any of the neutral objects you saw. Thinking about any of those pictures, please read each item below and then indicate how true each statement is for you.’ Participants then completed the four IES items relating to neutral images, and then repeated the process for the negative images. Data from the follow-up questionnaire was used to categorise participants into high vs low intrusion groups for between-group analyses (see below).

### Experimental Task and Design

#### Stimuli

Stimuli were selected from the International Affective Picture System (IAPS; [35]). Ten neutral (e.g. household objects) and ten highly negative and arousing images of injured people were selected as target stimuli. A further ten neutral and ten negative (equivalent to target images on valence and arousal ratings) images were selected from the same source as non-target distractor images for the visual processing condition. Ten non-affective control stimuli, consisting of black geometric shapes, were also constructed for use in a sensorimotor control condition.

#### Experimental task

The experiment used a modified version of the visual/verbal processing paradigm used by Hariri et al. [30], consisting of five conditions: negative and neutral visual, negative and neutral verbal, and sensorimotor control. In the negative and neutral visual conditions, participants were required to visually match a target stimulus to one of two distractor images. Trials consisted of one large negative or neutral IAPS target image, with two smaller distractor IAPS images presented below, one corresponding to the target, and the other serving as a non-target distractor image (similar in content to the target image). Participants were instructed to select which of the two images corresponded to the target by button press response. In the negative and neutral verbal conditions, participants were also presented with a negative or neutral IAPS target image, but were required to select one of two word labels ‘Natural’ or ‘Artificial’ that best corresponded to the target. The position of these words (left vs. right) was counterbalanced and pseudorandomised within participants. Participants were required to label the target image by pressing a button to choose one of the presented words. They were instructed to select the label that was most applicable to the image. In the neutral verbal condition, the expectation was that items such as animals and plants would be labeled ‘Natural’; and household or manmade objects as ‘Artificial’. In the negative verbal condition, participants were instructed to select the label that best corresponded to the cause of the injury depicted, with ‘natural’ including circumstances such as animal attack or natural disaster, and ‘artificial’ including motor vehicle accidents or attack with a weapon. In the sensorimotor control condition, participants were simultaneously presented with one larger target geometric shape and two smaller shapes beneath it. Participants used a button press to select which of the smaller shapes matched the target. This task was used as a baseline when processing the data, to eliminate brain activation associated with the sensorimotor responses needed to complete the task. The neutral visual and verbal conditions also served as a baseline comparison for investigating neural responses to intrusive images, since due to their nature only negative images were assumed to be likely to become intrusive.

#### Procedure

Participants underwent an fMRI scan while completing the visual/verbal task. A block design was used, consisting of five trials per block, with a total of two blocks per condition (10 blocks in total). The order of block presentations was counterbalanced between participants. Before each block, a slide was presented for four seconds which instructed participants to either ‘match pictures’ (visual and sensorimotor control conditions) or ‘label pictures’ (verbal condition). Each stimulus trial was presented for five seconds, with no inter-trial interval or rest periods, and each modeled block ran for 25 seconds (the total block time was 38 seconds, including instructions and stimulus ratings). After completing each block, participants provided a rating of their overall emotional reaction to the target stimuli on a 4-point Likert scale (1 = not at all negative, 2 = slightly negative, 3 = moderately negative, 4 = extremely negative). A slide was shown for nine seconds giving a visual representation of this rating scale, during which time the participants made their rating by pressing the corresponding button. There was no inter-stimulus interval between the stimulus slides and presentation of the rating scale.

Stimuli were presented via a computer monitor set up at the end of the scanner bore nearest to the participant’s head. This image was then viewed by the participant via a small adjustable mirror positioned outside of the head coil. Presentation of stimuli was controlled and logged via a computer running Presentation^®^ software (Version 14.7, Neurobehavioral Systems, www.neurobs.com). The experimental paradigm ran for 6.3 minutes, and took place during a scanning session of approximately 20 minutes, which included a structural scan, resting state scan and two non-affective functional MRI paradigms (i.e. global vs local attention task and sensory checkerboard task).

### Image Acquisition

fMRI data was collected using a Siemens Trio 3 Tesla scanner (Siemens, Erlangen, Germany) located at the Advanced Research and Clinical Highfield Imaging (ARCHI) facility at Royal North Shore Hospital in St Leonards, Sydney. Data was collected using gradient echo echo-planar imaging to depict blood oxygen level dependent (BOLD) activity. Twenty-nine ascending brain slices were acquired parallel to the AC-PC line (4mm thick with 1mm gap; effective thickness 5mm), 64 x 64 matrix: TR 2sec, TE 32ms, FOV of 240mm.

### Image Analysis

All fMRI data processing and analyses were performed using SPM8 (Wellcome Department of Imaging Neuroscience, London, UK; http://www.fil.ion.ucl.ac.uk/spm). The images underwent slice-time correction, realignment, reslicing, and normalization to the EPI template provided by SPM8. Slice timing correction used slice 16 as the reference slice, and used SPM’s Fourier phase shift interpolation. Motion correction used a rigid body spatial transformation, and registered to the mean image, using a two pass procedure. This registered the images to the mean image after the first alignment. Second degree B-spline interpolation was used in motion correction. The normalization process used affine regularization to SPM’s ICBM space template. Images were smoothed with an 8mm full-width half-maximum Gaussian kernel. Data was manually checked for alignment with the AC-PC line, and screened for excessive movement across scans (greater than 3mm or greater than 2 degrees rotation). Four participants were scanned but excluded from final analyses due to excessive movement during scanning, leaving a final sample size of thirty-eight participants.

### Statistical Analysis

Participants were divided into high intrusion and low intrusion groups based on the sum of their scores on the 4-item follow-up intrusion questionnaire. These items referred directly to the experience of intrusions relating to the negative images, and had a total score range of 0–16. Participants who scored 0 or 1 on these items were categorized into the low intrusion group, while participants who scored a total of 5 or higher summed across the 4-items were categorized as members of the high intrusion group. These upper and lower cutoff scores were selected *a priori* in order to delineate between high and low intrusion experiencers respectively, and exclude the mid-range group who did not fall clearly into either category in order to maximize between group comparisons.

One sample t-tests were conducted in SPM8 to investigate activations within the high and low intrusions groups. Subsequent two sample t-tests were conducted to compare activations in the high vs. low intrusion groups using the same contrasts as the within-group analysis. The contrasts used were: 1) Negative visual processing (relative to neutral): negative visual > neutral visual; 2) Negative verbal processing (relative to neutral): negative verbal > neutral verbal; 3) Negative verbal vs visual processing: negative verbal > negative visual and negative visual > negative verbal, with the sensorimotor control condition being used as an implicit baseline; 4) Neutral verbal vs neutral visual processing: neutral verbal > neutral visual, and neutral visual > neutral verbal. T-tests were conducted at the whole brain level, with a cluster based significance threshold of p < 0.05 FWE, and cluster threshold of 10 contiguous voxels.

Region of interest (ROI) analyses were also conducted to explicitly examine intrusion-related activation between high and low intrusions groups in regions previously associated with visual vs. verbal affective processing [30] and intrusions [28, 36]. Hariri et al. [30] reports that visual vs. verbal processing was distinguished by activity in the amygdala, anterior cingulate cortex, parahippocampal gyrus, fusiform gyrus, Broca’s area (inferior frontal gyrus, opercular and triangular parts), and the ventral prefrontal cortex; a number of these regions have also been associated with intrusions [28, 36]. We tested activity within these specific ROIs using standardized masks derived from the AAL atlas [37] within SPM8. We anticipated that differences in the BOLD signal between groups exhibiting high vs low intrusions to be relatively small. Indeed, previous fMRI studies have reported that the unpredictable nature of intrusions renders these types of memories resistant to efficient modelling via the BOLD signal [28]. We also expected the signal generated during the encoding of negative stimuli resulting in higher intrusions in this non-clinical sample to be relatively weaker than that which would be expected from clinically-relevant intrusions. Considering these limitations, and in accordance with previous fMRI studies of intrusions that implemented ROI analyses [28], coupled with the exploratory nature of this study, we adopted a significance threshold of p < 0.05 uncorrected, with a cluster threshold of 10 contiguous voxels to enable focused analysis on the expected small-scale variations in activity between high and low intrusions groups. Activation within these ROIs was compared in the high and low intrusion groups via 2 sample t-tests, using the same contrasts applied in the whole-brain analysis.

Additionally, independent samples t-tests were conducted to compare the behavioral (reaction time) and self-report measures between the high and low intrusion groups. Reaction time data was inspected for outliers more than two standard deviations above or below the mean. Outliers were replaced with the ±2SD value. In order to improve normality, the reaction time data was log10 transformed.

## Results

### High and Low Intrusion Group Characteristics

Thirteen participants were categorized into the low intrusion group (5 female, 8 male) with a mean intrusion score of 0.46 (*SD* = 0.52), and 13 participants were classified in the high intrusion group (9 female, 4 male) with a mean intrusion score of 10.08 (*SD* = 2.38). High and low intrusion groups did not differ in terms of age, or psychological measure scores (see Table 1). Seven participants in the high intrusion group and three in the low intrusion group had BDI-II scores indicative of mild to moderate depressive symptoms [31].

**Table 1:** Mean Participant Characteristics.

Measure	High Intrusion	Low Intrusion	t(df = 24)	*p*
Age	20.15 years (2.38)	19.15 years (1.38)	1.41	0.17
BDI-II	11.93 (6.95)	10.46 (9.91)	0.44	0.67
STAI	45.92 (7.08)	47.63 (2.94)	-0.80	0.43
IES	16.31 (8.78)	16.53 (12.17)	-0.05	0.96
VVIQ	58.31 (13.86)	57.82 (11.41)	0.09	0.93

Note. Standard deviations appear in parentheses.

### Behavioral Analyses

In their responses to the post-scanning questionnaire, participants rated the negative images as significantly more likely to be perceived as negative than the neutral images (t(24) = 22.913, p < 0.005), whilst also finding the neutral images more positive than the negative images (t(24) = -9.811, p < 0.005). There were no differences seen between the high intrusion and low intrusion groups on ratings of images (Table 2).

**Table 2:** Mean Post-Scanning Ratings.

	High Intrusion	Low Intrusion	t(df = 24)	*p*
‘How negative would most people find these?’ (negative images)	8.85 (1.41)	8.15 (1.07)	1.41	0.17
‘How negative would most people find these?’ (neutral images)	1.77 (1.54)	2.31 (1.60)	-0.88	0.39
‘How positive would most people find these?’ (negative images)	1.77 (1.64)	1.54 (1.13)	0.42	0.68
‘How positive would most people find these?’ (neutral images)	6.08 (2.63)	4.77 (1.83)	1.47	0.16

Note. Standard deviations appear in parentheses.

Participants were faster to match stimuli in the visual processing stimuli *M* = 3.12, *SD* = 0.07) and the sensorimotor control condition, compared with the verbal condition (*M* = 3.27, *SD* = 0.09), t(25) = -10.24, p < 0.001). Participants also responded more quickly to the sensorimotor control condition (*M* = 3.12, *SD* = 0.06) compared with the verbal condition t(25) = 8.56, p = < 0.001) but not the visual condition (t(25) = -0.13, p >0.05). There were no significant group effects between the high intrusion and low intrusion groups in terms of reaction time (visual: t(24) = 0.66, p > 0.05; verbal: t(24) = 0.-0.25, p > 0.05; sensorimotor control: t (24) = -0.55, p > 0.05). A two-way mixed model ANOVA failed to find any group x condition interactions for reaction time (F(2,48) = 0.60, p > 0.05).

### Imaging Analyses

#### Whole brain analyses

**Fig 1:**
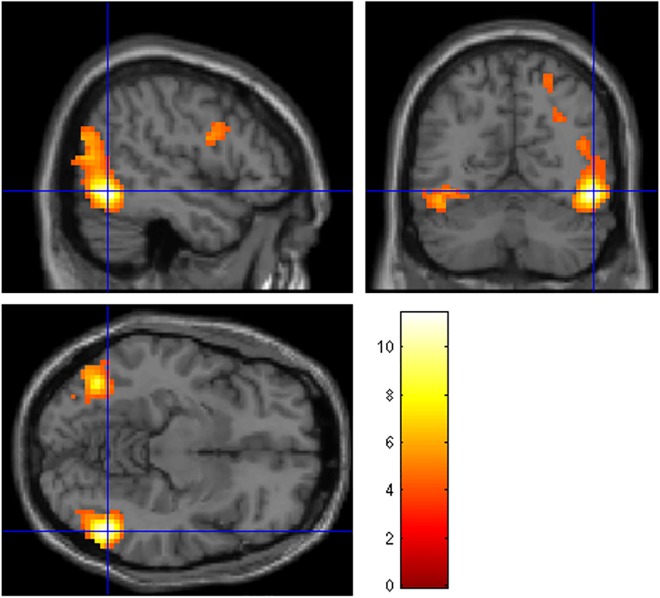
Regions of BOLD activation during the contrast negative visual > neutral visual for the High Intrusion group. Crosshairs indicate peak activity in the inferior temporal gyrus (FWE 0.05 corrected at the cluster level, cluster threshold of 10 contiguous voxels).

**Fig 2:**
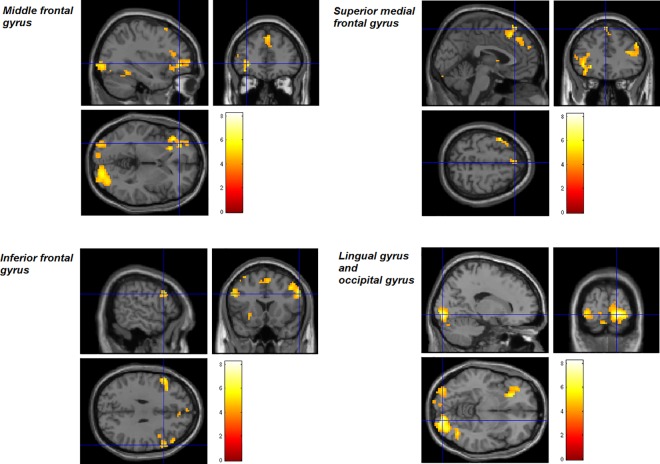
Regions of BOLD activation during the contrast negative verbal > neutral verbal for the High Intrusion group. Crosshairs indicate peak activity in the cluster described in the legend (FWE 0.05 corrected at the cluster level, cluster threshold of 10 contiguous voxels).

**Fig 3:**
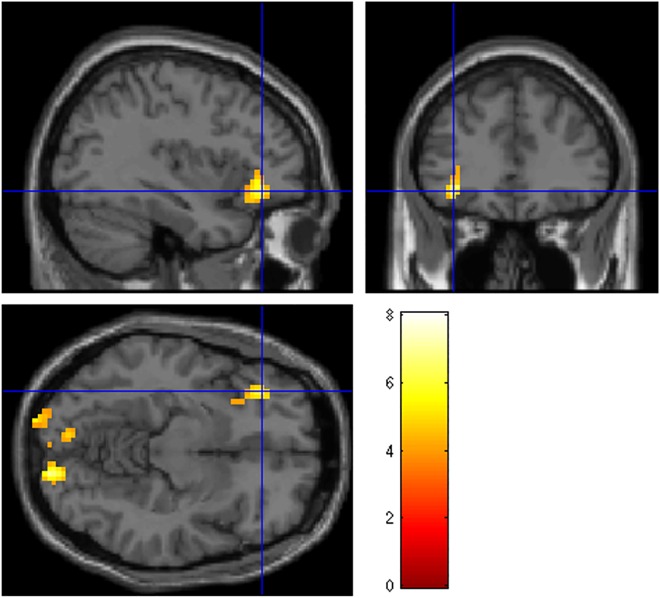
Regions of BOLD activation during the contrast negative verbal > negative visual for the High Intrusion group. Crosshairs indicate inferior frontal gyrus activity (FWE 0.05 corrected at the cluster level, cluster threshold of 10 contiguous voxels).

Within-group one sample t-tests in the high intrusion group (Table 3) revealed significant clusters of BOLD activity during negative visual processing (vs neutral) in visual processing areas including the right inferior temporal gyrus and fusiform gyrus (Fig 1), while the negative verbal condition (vs. neutral verbal; Fig 2) activated a variety of left lateralized regions, including the left lingual gyrus, left middle frontal gyrus, right inferior frontal gyrus (opercular part), left superior medial frontal gyrus and left middle occipital gyrus. Furthermore, the high intrusions group demonstrated greater BOLD signal during negative verbal processing in the right calcarine gyrus; left inferior frontal gyrus, orbital part, and left lingual gyrus in the negative verbal > negative visual condition (Fig 3). No significant activations were seen in the negative visual > negative verbal, neutral verbal > neutral visual or neutral visual > neutral verbal conditions.

**Table 3:** High Intrusion group, whole brain, p < 0.05 FWE corrected at the cluster level, within samples one-sample t-test.

Contrast	Region	MNI coordinates of peak activation (x, y, z)	Cluster size	Cluster p corr (FWE 0.05)	*t*
Negative Visual > Neutral Visual	Inferior Temporal Gyrus R	51–61–10	1069	0.000	11.42
	Fusiform Gyrus L	-39–55–16	606	0.000	8.76
Negative Verbal > Neutral Verbal	Lingual Gyrus L	18–91–7	688	0.000	8.25
	Middle Frontal Gyrus L	-30 41–1	297	0.000	6.76
	Inferior Frontal Gyrus, Opercular Part L	60 17 32	275	0.000	6.05
	Middle Frontal Gyrus R	-42 8 53	218	0.001	6.01
	Superior Medial Frontal Gyrus L	0 32 62	109	0.024	6.16
	Middle occipital Gyrus L	-33–91–4	117	0.018	5.30
Negative Verbal > Negative Visual	Calcarine Gyrus R	15–97–7	107	0.018	8.05
	Inferior Frontal Gyrus, Orbital Part L	-36 35–10	81	0.049	6.59
	Lingual Gyrus L	-12–100–16	137	0.006	6.57
Negative Visual > Negative Verbal	No significant activations				

**Fig 4:**
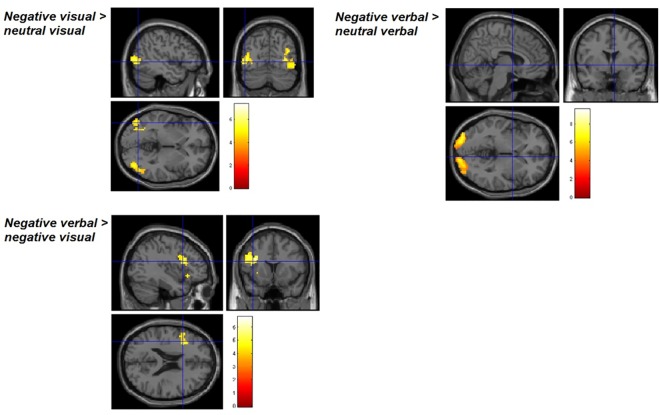
Regions of BOLD activation for the Low Intrusion group (FWE 0.05 corrected at the cluster level, cluster threshold of 10 contiguous voxels).

Similarly, the low intrusion group (Table 4) showed significant clusters of activation for all contrasts except negative visual > negative verbal. Both the negative visual and negative verbal conditions activated occipital regions relative to neutral (Fig 4), while the negative verbal > negative visual condition showed activation in the triangular part of the left inferior frontal gyrus (Fig 4). No significant activations were seen in the neutral verbal > neutral visual or neutral visual > neutral verbal conditions. Significant activations were not seen in any contrast investigating deactivations (i.e. neutral > negative) in either the high or low intrusion group. Two sample t-tests comparing BOLD activations between the high intrusion and low intrusion groups did not yield any significant clusters at the whole-brain level.

**Table 4:** Low Intrusion group, whole brain, p < 0.05 FWE corrected at the cluster level, within samples one-sample t-test.

Contrast	Region	MNI coordinates of peak activation (x, y, z)	Cluster size	Cluster p corr (FWE 0.05)	*t*
Negative Visual > Neutral Visual	Middle Occipital Gyrus L	-42–82 17	231	0.000	6.26
	Inferior Occipital Gyrus R	42–76–7	484	0.000	6.15
Negative Verbal > Neutral Verbal	Middle Occipital Gyrus L	-21–100 5	131	0.000	9.60
	Inferior Occipital Gyrus L	33–97–7	335	0.000	7.61
Negative Verbal > Negative Visual	Inferior Frontal Gyrus, Triangular Part L	-36 17 23	196	0.001	6.74
Negative Visual > Negative Verbal	No significant activations				

#### ROI analyses: High vs low intrusions group comparisons

During the negative verbal condition, the high intrusion group displayed significantly greater activations than the low intrusion group in the left amygdala, bilateral parahippocampal gyrus, bilateral ACC (peak activation in the left dorsal ACC (dACC), extending bilaterally to the right ACC), the right inferior frontal gyrus, opercular part, and left inferior frontal gyrus, triangular and orbital parts (Table 5).

**Table 5:** Region of interest analysis: 2 sample t-test High Intrusion > Low intrusion Group, Negative Verbal > Neutral Verbal, uncorrected 0.05.

Region	MNI coordinates of peak activation (x, y, z)	Cluster size	Cluster p corr	voxel p uncorr	*t*
Amygdala L	-27 2–19	15	0.442	0.009	2.56
ACC L (peak in left side, but cluster extends bilaterally to ACC R)	-1 8 29	123	0.513	0.003	3.06
Parahippocampal Gyrus R	18–34–10	17	0.865	0.002	3.18
Parahippocampal Gyrus R	15–1–22	17	0.865	0.004	2.89
Inferior Frontal Gyrus Triangular Part L	-30 29–1	53	0.907	0.003	3.04
Inferior Frontal Gyrus, Opercular Part R	45 11 38	37	0.935	0.005	2.79
Inferior Frontal Gyrus, Orbital Part L	-27 32–4	105	0.638	0.000	4.09

During the negative visual condition, the high intrusion group showed significantly greater activations than the low intrusion group in the left inferior frontal gyrus, opercular part, and the right inferior frontal gyrus, triangular and orbital parts (Table 6).

**Table 6:** Region of interest analysis: 2 sample t-test High Intrusion > Low intrusions Group Negative Visual > Neutral Visual, uncorrected 0.05.

Region	MNI coordinates of peak activation (x, y, z)	Cluster size	Cluster p corr	voxel p uncorr	*t*
Inferior Frontal Gyrus Opercular Part L	-51 14 20	396	0.462	0.002	3.11
Inferior Frontal Gyrus Triangular Part R	33 26 26	20	0.991	0.005	2.82
Inferior Frontal Gyrus Triangular Part R	36 32 11	60	0.963	0.005	2.79

## Discussion

The key findings of this study suggest that healthy participants who reported a higher level of intrusions following exposure to negative images, displayed activations in the amygdala, bilateral ACC and parahippocampal gyrus during verbal encoding, compared to those who experienced low levels of subsequent intrusions. The high intrusion group also showed greater activity in dorsomedial (dmPFC) and dorsolateral PFC (dlPFC), inferior frontal gyrus and occipital regions during negative verbal processing compared to neutral. In contrast, the low intrusions group displayed singular activation in sensory occipital regions during verbal processing. Activity during negative visual processing was less pronounced, with the high intrusion group displayed activations in temporal regions, whereas the low intrusion group again showed activations in occipital sensory regions. When directly comparing activations which were greater during negative verbal processing than negative visual processing, activity was seen in the inferior frontal gyrus in both the high and low intrusion groups. No significant activations were observed for either group during negative visual processing relative to negative verbal processing.

These results were partly in line with our hypotheses, which predicted activations in amygdala and MTL structures, ACC and mPFC during the encoding of negative cues that resulted in higher-reported intrusions, and with ACC activation specifically being associated with verbal processing. Results diverged from those hypothesised in that activations associated with intrusions were seen specifically in the verbal processing, rather than the visual processing, conditions. Results also diverged from hypotheses derived from previously reported visual vs verbal processing studies [30] findings, which specifically reported enhanced amygdala activity during visual not verbal processing. However, this previous study did not investigate intrusive memories, which may explain the differences seen in the results.

The high intrusions group activated key frontal regions during negative verbal processing that have been previously associated with intrusive memories–including the dmPFC, inferior frontal gyrus [28] and dlPFC [36]. These particular regions may be involved in enhanced encoding of negative cues, and with activation predicting later retrieval. Of these frontal regions, the dmPFC is associated with fear processing, emotional appraisal and expression [38, 39], which is consistent with the possibility that emotional processing enhances encoding of intrusions. Inhibitory processes may also contribute to the development of intrusions, in line with thought suppression theories of intrusions [8]. Thus it is of interest that the dlPFC and inferior frontal gyrus have been implicated in cognitive control processes and the suppression of emotional responses [26, 40–43], with activations in these regions potentially suggesting support for these theories. However, further research is needed to clarify the possible role of inhibition in the encoding of intrusions. In addition, the high intrusion group also showed activations in Broca’s area (inferior frontal gyrus, opercular and triangular parts) during verbal conditions (most likely because of verbal processing invoked by the task). Both high and low intrusions groups demonstrated activation within sensory occipital and temporal regions during negative verbal processing, since even the verbal conditions involved a strong visual component.

Results from the ROI analyses lend marginal support to the conclusions drawn from the whole brain analysis. Significant activation was observed in the negative verbal condition in regions critical to emotion processing including the amygdala, dACC and parahippocampal gyrus, as well as in the inferior frontal gyrus. In the negative visual condition, activations were seen in the inferior frontal gyrus. However, it should be kept in mind that these ROI analyses were exploratory and a reduced threshold was adopted. Further research is necessary to confirm these findings in a larger sample.

How do we understand these results in the context of current models of intrusive memories? The activation of more inhibitory networks in those participants who displayed intrusions may reflect compensatory responses by those people because they were particularly distressed by the information they were encoding. Thought suppression theory posits that intrusions occur because of excessive suppression of target information, which ironically results in preferential monitoring of internal representations of these memories, thereby leading to involuntary occurrences of these memories [8]. There is much evidence supporting this model of intrusion memories [44, 45], and the current findings may reflect the neural responses that occur at the time of encoding which reflect this inhibitory response to suppress full processing. It is also possible that participants who experienced intrusions may have personally related to the experience of the injured victims shown in the negative images, with development of intrusions resulting from the enhanced emotional processing involved in an empathic response, resulting in the greater activation in the inferior frontal gyrus.

Contrary to prediction, this study did not find the visual-based processing condition to be associated with activity in regions hypothesized to be involved in intrusions. Rather, brain activity was observed in regions relevant to intrusions (amygdala, ACC, parahippocampal gyrus) specifically during the verbal processing condition. These very regions are also critical to the development of emotional memories, including contextual memory encoding, and are implicated in the experience of anxiety, fear and functional disruptions that appear to underpin mechanisms of PTSD [46, 47]. The specificity of findings to the verbal processing conditioning could be due to the need for more detailed assessment of the negative content of the image in order to perform the verbal task, resulting in greater activation in these emotional memory regions during encoding for the high intrusions group. An alternative explanation is that competition for intrusion-protective verbal resources leads to greater intrusive activity due to increased perceptual processing of the negative visual image [18]. In the current study, the nature of the task was such that more effort was likely required to respond to images in negative verbal, than visual, condition because the stimuli and associated labels were somewhat ambiguous. Reaction time data supports this, with faster responses in visual conditions than in verbal conditions. The visual condition was relatively easy to complete because participants could readily match images without fully processing the meaning of the image. Current models of intrusions allude to the importance of sufficient cognitive resources being available to encode and contextualize the unpleasant information in such a way that it can be fully understood within one’s normal memory base [48]. If these resources are insufficient, then one reverts to focusing on perceptual features of the experience, which can then contribute to subsequent intrusions. It is also possible that the ease with which the visual condition could be completed allowed for avoidance, where participants spent less time looking at the negative images in the visual condition as a means of emotion regulation.

A limitation of the current study is the difference in difficulty between the visual and verbal conditions. Future research could improve upon the paradigm used by making the stimuli in each trial of the visual condition more similar, thereby equating the cognitive resources required to complete the visual and verbal tasks, and helping to eliminate the possibility of participants attempting to avoid processing the negative aspects of images in the visual condition. Another limitation which must be kept in mind is that the whole brain results discussed in this paper were seen in within-groups analysis only; no significant between-group activations were observed at the threshold implemented. Although these results give some preliminary indications of the brain regions associated with the encoding of intrusive memories under visual and verbal conditions, the lack of significance in the between groups results may have been due to the size of the sample used. The ROI group comparisons implemented a more lenient threshold to determine significant activations. These findings therefore need to be confirmed using different experimental paradigms and larger sample sizes. Further research using larger samples would be useful in confirming the results of this study at a between-groups level. In addition, the use of a block design in this study did not allow for the identification of the specific images were experienced intrusively. In order to gain stronger BOLD signal associated with intrusions, further research should be considered using event-related designs, which would allow identification of activations associated with encoding of specific intrusive images.

The current findings provide initial evidence that intrusive memories are related to activation of regions involved in emotion processing, emotional memory encoding and emotional and behavioral inhibition during effortful verbal processing at encoding, but not under visual processing conditions. This novel finding is interesting because it does not align with models that argue that perceptually-driven processing is more closely associated with intrusive memories. Rather, it may be that increased resources at encoding due to a higher demand verbal task, and resulting activation of emotional and cognitive networks, could lead to compensatory perceptual based consolidation, and associated increases in subsequent intrusive memories. It is also important to note that whereas one school of thought posits that development of intrusive memories is centrally linked to encoding processes, other theories emphasize factors associated with consolidation and retrieval phases of memory. It is important for future neuroimaging studies to investigate the neural networks associated with unintentional recall of memories at each of these phases, in addition to encoding processes as investigated in this study.

## Supporting Information

S1 Dataset Participant characteristics and behavioral data of intrusive memories (SAV)
